# An investigation of the distribution of gaze estimation errors in head mounted gaze trackers using polynomial functions

**DOI:** 10.16910/jemr.11.3.5

**Published:** 2018-06-30

**Authors:** Diako Mardanbegi, Andrew T. N. Kurauchi, Carlos H. Morimoto

**Affiliations:** Department of Management Engineering, Technical University of Denmark, Denmark; Department of Computer Science (IME), University of Sao Paulo, Sao Paulo, Brazil

**Keywords:** Eye Movement, eye tracking, saccades, microsaccades, antisaccades, smooth pursuit, scanpath, convergence, attention

## Abstract

Second order polynomials are commonly used for estimating the point-of-gaze in headmounted eye trackers. Studies in remote (desktop) eye trackers show that although some non- standard 3rd order polynomial models could provide better accuracy, high-order polynomials do not necessarily provide better results. Different than remote setups though, where gaze is estimated over a relatively narrow field-of-view surface (e.g. less than 30×20 degrees on typical computer displays), head-mounted gaze trackers (HMGT) are often desired to cover a relatively wider field-of-view to make sure that the gaze is detected in the scene image even for extreme eye angles. In this paper we investigate the behavior of the gaze estimation error distribution throughout the image of the scene camera when using polynomial functions. Using simulated scenarios, we describe effects of four different sources of error: interpolation, extrapolation, parallax, and radial distortion. We show that the use of third order polynomials result in more accurate gaze estimates in HMGT, and that the use of wide angle lenses might be beneficial in terms of error reduction.

## Introduction

Monocular video-based head mounted gaze trackers use at least one camera to capture the eye image and another to capture the field-of-view (FoV) of the user. Probably due to the simplicity of regression-based me-thods when compared to model-based methods [ 10 ], regression-based methods are commonly used in head-mounted gaze trackers (HMGT) to estimate the user’s gaze as a point within the scene image, despite the fact that such methods do not achieve the same accuracy levels of model-based methods.

In this paper we define and investigate four different sources of error to help us characterize the low performance of regression-based methods in HMGT. The first source of error is the inaccuracy of the gaze mapping function in interpolating the gaze point (e_int_) within the calibration box, the second source is the limitation of the mapping function to extrapolate the results outside the calibration box required in HMGT (e_ext_), the third is the misalignment between the scene camera and the eye known as parallax error (e_par_), and the fourth error source is the radial distortion in the scene image when using a wide angle lens (e_dis_).

Most of these sources of error have been investigated before independently. Cerrolaza et al. [ 8 ] have studied the performance, based on the interpolation error, of different polynomial functions using combinations of eye features in re- mote eye trackers. Mardanbegi and Hansen [ 12 ] have described the parallax error in HMGTs using epipolar geometry in a stereo camera setup. They have investigated how the pattern of the parallax error changes for different camera configurations and calibration distances. However, no experimental result was presented in their work showing the actual error in a HMGT. Barz et al. [ 2 ] have proposed a method for modeling and predicting the gaze estimation error in HMGT. As part of their study, they have empirically investigated the effect of extrapolation and parallax error independently. In this paper, we describe the nature of the four sources of error introduced above in more details providing a better understanding of how these different components contribute to the gaze estimation error in the scene image. The rest of the paper is organized as follows: The simulation methodology used in this study is described in the first section and the next section describes related work regarding the use of regression-based methods for gaze estimation in HMGT. We then propose alternative polynomial models and compare them with the existing models. We also show how precision and accuracy of different polynomial models change in different areas of the scene image. Section Parallax Error describes the parallax error in HMGT and its following section investigates the effect of radial distortion in the scene image on gaze estimation accuracy. The combination of errors caused by different factors is discussed in Section Combined Error and we conclude in Section Conclusion.

## Simulation

All the results presented in the paper are based on simulation and the proposed methods are not tested on real setups. The simulation code for head-mounted gaze tracking that was used in this paper was developed based on the eye tracking simulation framework proposed by Böhme, Dorr, Graw, Martinetz, & Barth [ 6 ].

The main four components of a head-mounted eye tracker (eye globe, eye camera, scene camera and light source) are modeled in the simulation. After defining the relationship between these components, points can be projected from 3D to the camera images, and vice versa.

Positions of the relevant features in the eye image are computed directly based on the geometry between the components (eye, camera and light) and no 3D rendering algorithms and image analysis are used in the simulation. Pupil center in the eye image is obtained by projecting the center of pupil into the image and no ellipse fitting is used for the tests in this paper. The eyeball can be oriented in 3D either by defining its rotation angles or by defining a fixation point in space. Fovea displacement and light refraction on the surface of the cornea are considered in the eye model.

The details of the parameters used in the simulation are described in each subsequent section.

## Regression-based methods in HMGT

The pupil center (PC) is a common eye feature used for gaze estimation [ 10 ]. Geometry-based gaze estimation methods [ 9,15 ] mostly rely on calculating the 3D position of the pupil center as a point along the optical axis of the eye. Feature-based gaze estimation methods, on the other hand, directly use the image of the pupil center (its 2D location in the eye image) as input for their mapping function.

Infrared light sources are frequently used to create corneal reflections, or glints, that are used as reference points. When combined, the pupil-center and glint (first Purkinje image [ 13 ]) forms a vector (in the eye image) that can be used for gaze estimation instead of the pupilcenter alone. In remote eye trackers, the use of the pupilglint vector (PCR) improves the performance of the gaze tracker for small head motions [ 16 ]. However, eye movements towards the periphery of the FoV are often not tolerated when using glints as the reflections tend to fall off the corneal surface. For the sake of simplicity, in the following, we use pupil center instead of PCR as the eye feature used for gaze mapping.

**Figure 1.**
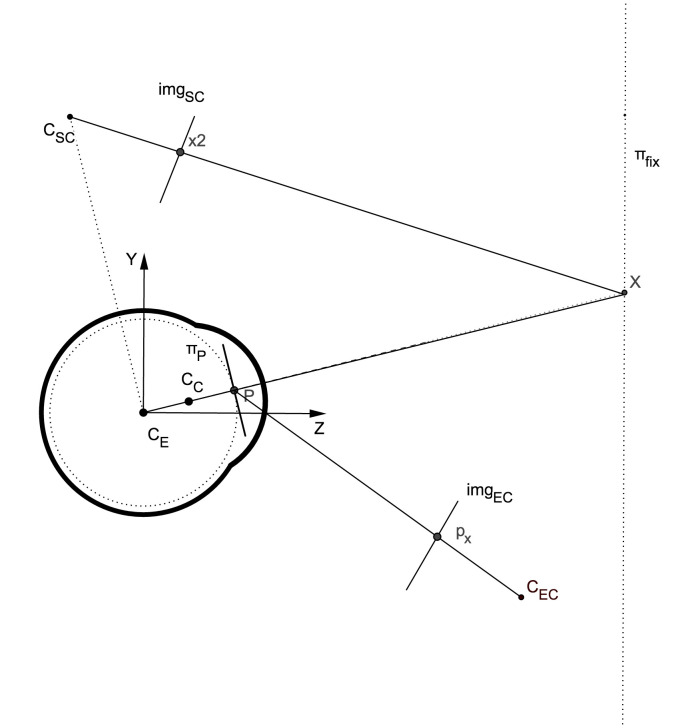
Sagittal view of a HMGT

Figure 1 illustrates the general setup for a pupil-based HMGT consisting of 3 components: the eye, the eye camera, and the scene camera. Gaze estimation essentially maps the position of the pupil center in the eye image (p_x_) to a point in the scene image (x) when the eye is looking at a point (X) in 3D.

Interpolation-based (regression-based) methods have been widely used for gaze estimation in both commercial eye trackers and research prototypes in remote (or desktop) scenarios [ 7,8,17 ]. Compared to geometry-based methods [ 10 ], they are in general more sensitive to head movements though they present reasonable accuracy around the calibration position, they do not require any calibrated hardware (e.g. camera calibration, and predefined geometry for the setup), and their software is simpler to implement. Interpolation-based methods use linear or non-linear mapping functions (usually a first or second order polynomial). The unknown coefficients of the mapping function are fitted by regression based on correspondence data collected during a calibration procedure. It is desirable to have a small number of calibration points to simplify the calibration procedure, so a small number of unknown coefficients is desirable for the mapping function.

In a remote gaze tracker (RGT) system, one may assume that the useful range of gaze directions is limited to the computer display. Performance of regression-based methods that map eye features to a point in a computer display have been well studied for RGT [ 5,8,18 ]. Cerrolaza et al. [ 8 ] present an extensive study on how different polynomial functions perform on remote setups. The maximum range of eye rotation used in their study was about (16° × 12°) (looking at a 17 inches display at the distance 58 cm). Blignaut [ 4 ] showed that a third order polynomial model with 8 coefficients for S_x_ and 7 coefficients for S_y_ provides a good accuracy (about 0.5°) on a remote setup when using 14 or more calibration points.

However, performance of interpolation-based methods for HMGT have not yet been thoroughly studied. The mapping function used in a HMGT maps the eye features extracted from the eye image to a 2D point in the scene image that is captured by a front view camera (scene camera) [ 11 ]. For HMGT it is common to use a wide FoV scene camera (FoV > 60°) so gaze can be observed over a considerably larger region than RGT. Nonetheless, HMGTs are often calibrated for only a narrow range of gaze directions. Because gaze must be estimated over the whole region covered by the scene camera, the polynomial function must extrapolate the gaze estimate outside the bounding box that contains the points used for calibration (called the calibration box). To study the behavior of the error inside and outside the calibration box, we will refer to the error inside the box as interpolation error and outside as extrapolation error. The use of wide FoV lenses also increases radial distortions which affect the quality of the scene image.

On the other hand, if the gaze tracker is calibrated for a wide FoV that spans over the whole scene image, it will increase the risk of poor interpolation. This has to do with the significant non-linearity that we get in the domain of the regression function (due to the spherical shape of the eye) for extreme viewing angles. Besides the interpolation and extrapolation errors, we should take into account the polynomial function is adjusted for a particular calibration distance while in practice the distance might vary significantly during the use of the HMGT.

## Derivation of alternative polynomial models

To find a proper polynomial function for HMGTs and to see whether the commonly used polynomial model is suitable for HMGTs, we will use a systematic approach similar to the one proposed by Blignaut [ 4 ] for RGTs. The systematic approach consists of considering each dependent variable S_x_ and S_y_ (horizontal and vertical components of the gaze position on the scene image) separately. We first fix the value for the independent variable P_y_ (vertical component of the eye feature in our case, pupil center or PCR - on the eye image) and vary the value of P_x_ (horizontal component of the eye feature on the eye image) to find the relationship between S_x_ and P_x_. Then the process is repeated fixing P_x_ and varying P_y_ to find the relationship between coefficients of the polynomial model and P_y_.

We simulated a HMGT with a scene camera described in Table 2. A grid of 25×25 points in the scene image (the whole image covered) are back-projected to fixation points on a plane at 1 m away from C_E_ and the corresponding pupil position is obtained for each point. We run the simulation for 9 different eyes defined by combining 3 different values for each of the parameters shown in Table 1 (3 parameters and ±25% of their default values). We extract the samples for two different conditions, one with pupil center and the second condition with pupil-glint vector as our independent variable.

**Table 1.** Default eye measures used in the simulation

r_cornea	7.98 mm
Horizontal fovea offset (α)	6°
Verical fovea offset (β)	2°

**Table 2.** Default configuration for the cameras and the light source used in the simulation. All measures are relative to the world coordinate system with the origin at the center of the eyeball (C_E_) (see Figure 1). The symbols R and Tr stands for rotation and translation respectively.

Scene camera	FoV = H : 65° × V : 40°, R = (pan, tilt, yaw) = (0, 0, 0), Tr = (10mm, 30mm, 35mm), no radial distortion, res=(1280 × 768)
Eye camera	focal length: providing an eye image with W_eye_/W_img_= 90% where W_eye_ is the horizontal dimension of the eye area in the image and W_img_ is the image width. R: satisfying the assumption of camera being towards eyeball center, Tr = (0mm, -10mm, 60mm), res=(1280 × 960)
Light source	Tr = (0, 0, 60mm)

**Figure 2.**
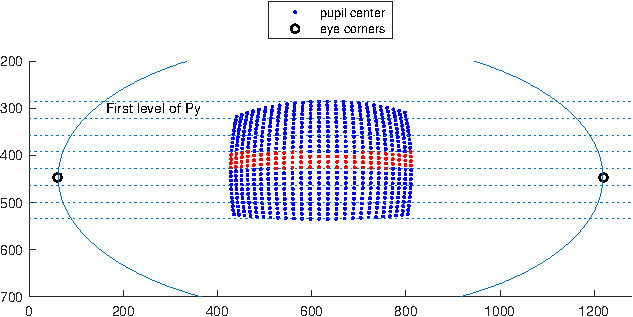
Virtual eye socket showing 625 pupil centers. Each center corresponds to an eye orientation that points the optical-axis of the eye towards a scene target on a plane 1 m from the eye, and each point on the plane corresponds to an evenly distributed 25 × 25 grid point in the scene camera. Samples were split into 7 groups based on their P_y_ values by discretizing the Y axis. Samples in the middle group are shown in a different color.

Figure 2 shows a virtual eye socket and the pupil center coordinates corresponding to 625 (grid of 25×25) target points in the scene image for one eye model. Let X and Y axis correspond to the horizontal and vertical axis of the eye camera respectively. To express S_x_ in terms of P_x_ we need to make sure the other variable P_y_ is kept constant. However, we have no control on the pupil center coordinates and even taking a specific value for S_y_ in the target space (as it was suggested in [ 4 ]) will not result in a constant P_y_ value. Thus, we split the sample points along the Y axis into 7 groups based on their P_y_ values by discretizing the Y axis. 7 groups give us enough samples in each group that are distributed over the X axis. This grouping makes it possible to select only the samples that have a (relatively) constant P_y_.

By keeping the independent variable P_y_ within a specific range (e.g., from pixel 153 to 170, which roughly corresponds to the gaze points at middle of the scene image), we can write about 88 relationships for S_x_ in terms of P_x_.

**Figure 3.**
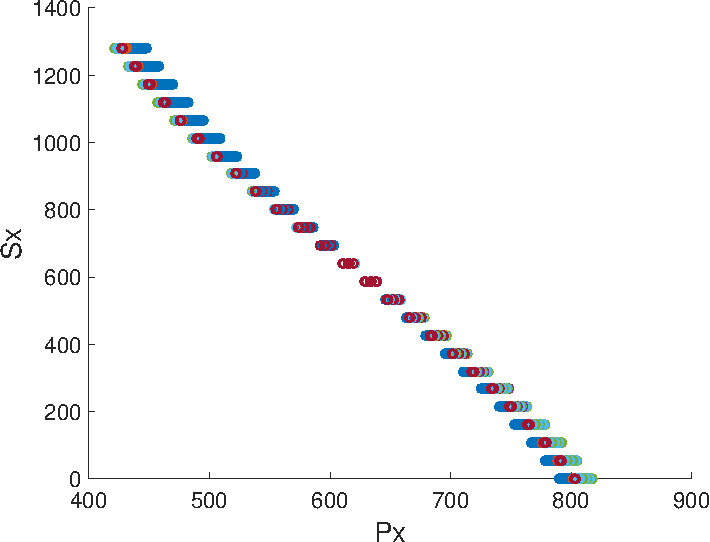
Relationship between the input P_x_ (pupil_x_) and output (S_x_). Different curves show the result for different parameters in the eye models.

Figure 3 shows this relationship which suggests the use of a third order polynomial with the following general form:

**(1):**
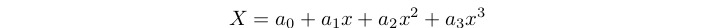


**Figure 4.**
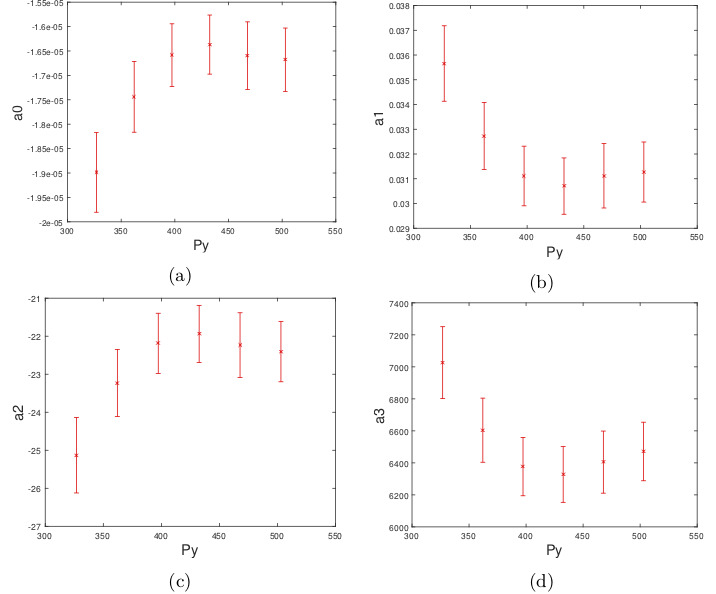
Relationship between the coeffcients a_i_ of the regression function S_x_ against Y coordinate of the pupil center.

We then look at the effect of changing the variable P_y_ on coeffcients a_i_. To keep the distribution of independent variable P_y_ on coefficients ai. To keep the distribution of samples across the X axis uniform when changing the P_y_ level, we skip the first level of P_y_ (Figure 2). The changes of _a_i against 6 levels of P_y_ are shown in Figure 4. From the figure we can see that relationship between coefficients a_i_ and the Y coordinate of the pupil center is best represented by a second order polynomial:

**(2):**
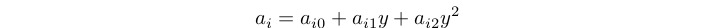


The general form of the polynomial function for S_x_ is then obtained by substituting these relationships into (Eq.1) which will be a third order polynomial with 12 terms:

**(3):**
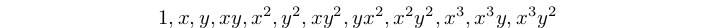


**Figure 5.**
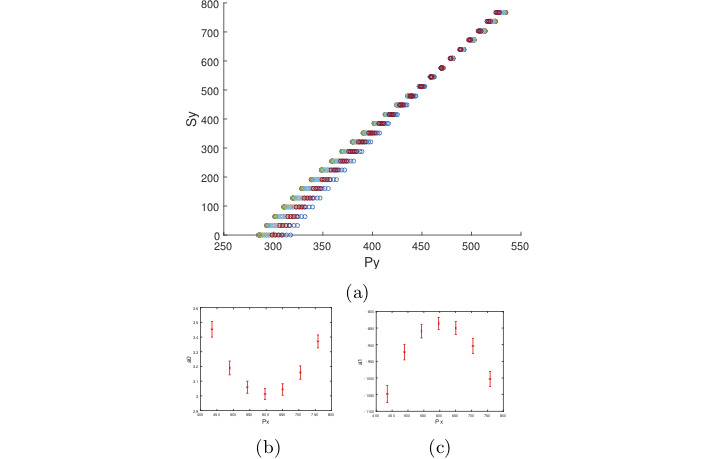
(5a) Relationship between the regression function S_y_ against the Y coordinate of the pupil center. (5b & 5c) Relationship between the coeffcients a_i_ of S_y_ against P_x_

We follow a similar approach to obtain the polynomial function for S_y_. Figure 5a shows the relationship between S_y_ and the independent variable P_y_ from which it can be inferred that a straight line should fit the samples for 27 different eye conditions. Based on this assumption we look at the relationship between the two coefficients of the quadratic function and P_x_. The result is shown in Figure 5b & 5c which suggests that both coefficients could be approximated by second order polynomials resulting that S_y_ to be a function with the following terms:

**(4):**
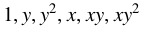


To determine the coefficients for S_x_ at least 12 calibration points are required, while S_y_ only requires 6. In practice the polynomial functions for S_x_ and S_y_ are determined from the same data. As at least 12 calibration points will already be collected for S_x_, a more complex function could be used for S_y_. In the evaluation section we show results using the same polynomial function (Eq.3) for both S_x_ and S_y_. However, to better characterize the simulation results we first introduce the concept of interpolation and extrapolation regions in the scene image.

### Interpolation and extrapolation regions

**Figure 6.**
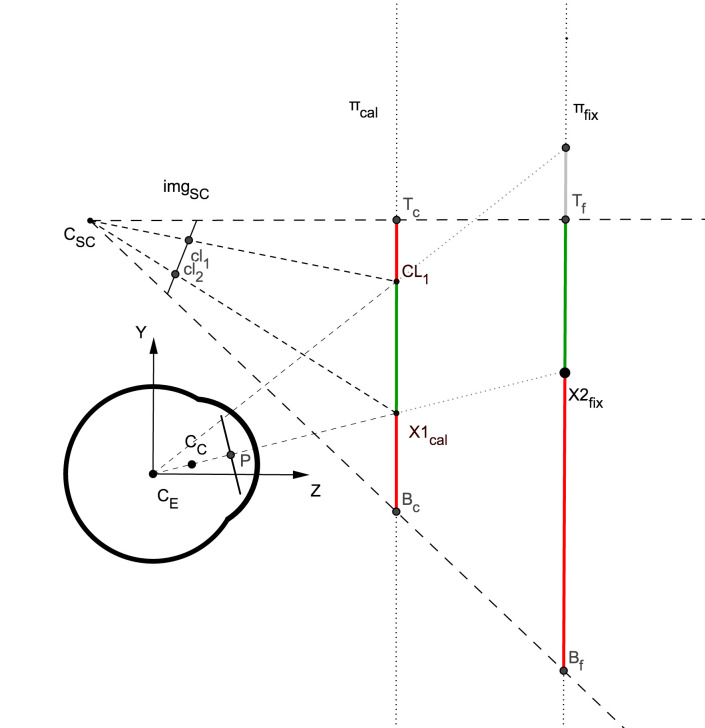
Sagittal view of a HMGT

Gaze mapping calibration is done by taking corresponding sample points from the range and the domain. This is usually done by asking the user to look at a set of co-planar points at a fixed distance (a.k.a calibration plane). For each point, the corresponding position in the scene image and the pupil position in the eye image are stored. Any gaze point inside the bounding box of the calibration pattern (the calibration box) will be interpolated by the polynomial function. If a gaze point is outside the calibration box it will be extrapolated. This is illustrated in Figure 6, where T_c_B_c_ is the area in the calibration plane (π_cal_) that is visible in the scene image. Let CL₁ and CL₁ be the edges of the calibration pattern. Any gaze position in π_cal_ within the range from T_c_ to CL₁ or from CL₁ to B_c_ will be extrapolated by the polynomial function. These two regions in the calibration plane are marked in red in the figure. We can therefore divide the scene image into two regions depending on whether the gaze point is interpolated (calibration box) or extrapolated (out of the calibration box).

In order to be able to express the relative coverage of these two regions on the scene image, we use a measure similar to the one suggested by Barz et al.[ 2 ]. We define S_int_ as the ratio between the interpolation area and the total scene image area:

**(5):**
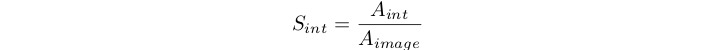


We also refer to S_int_ as the interpolation ratio in the image.

### Gaze estimation error when changing fixation depth

From now on, we refer to any fixation point in 3D by its distance from the eye along the Z axis. Therefore, we define fixation plane as the plane that includes the fixation point and is parallel to the calibration plane. T_f_B_f_ in Figure 6 shows the part of the fixation plane that is visible in the image. We can see that the interpolated (green) and extrapolated (red) regions in the scene image would change when the fixation plane π_fix_ diverges from the calibration plane. Projecting the red segment on the fixation plane π_fix_ into the scene image will define a larger extrapolated area in the image. Accordingly, the interpolated region in the image gets smaller when the fixation plane goes further away. Therefore, the interpolation ratio that we get for the calibration plane (S^cal^_int_ ) is not necessarily equal to the interpolation ratio that we have for different depths. Not only the size of the interpolation area changes when changing the fixation depth, but also the position of the interpolation region changes in the image.

Figure 6 illustrates a significant change in the value of S_int_ for a small variation of fixation distance which happens at very close distances to the eye. We simulate a HMGT with the simplified eye model (described in Table 1) and a typical scene camera configuration described in Table 2 to see whether changes of S_int_ are significant in practice. The result is shown in Table 3 for different fixation distances on a gaze tracker calibrated at distances 0.6 m and 3.0 m. We assume that the calibration pattern covers about 50% of the image (S^cal^_int_ = 0.5).

**Table 3.** S_int_ at different fixation distances for two different calibration distances

	d_cal_=0.6 m	d_cal_=3 m
0.6 m	48.8%	47%
1 m	45.3%	48.8%
3 m	42%	49.9%
5 m	41%	49.3%

The amount of change in the expansion of the interpolation region depends on the configuration of the camera and the epipole location in the scene image which is described by epipolar geometry (see Section Parallax Error). However, the result shows that for an ordinary camera setup, these changes are not significant.

### Practical grid size and distance for calibration

There are different ways to carry out calibration in HMGTs. The common way is to ask the user to look at different targets located at a certain distance from the eye (calibration distance) and recording sample points from the eye and scene images while user is fixating on each target. Target points in the scene image could be either marked and picked manually by clicking on the image (direct pointing) or it could be detected automatically (indirect pointing) using computer-vision-based methods. The targets are usually markers printed out on papers and attached to a wall or are displayed on a big screen (or projectors) in front of the user during calibration.

Alternatively, targets could be projected by a laser diode [ 1 ] allowing the calibration pattern to cover a wider range of the field of view of the scene camera. However, the practical size (angular expansion) for the calibration grid is limited to a certain range of the FoV of the eye. The further the calibration plane is from the subject the smaller the angular expansion of the calibration grid will be. Calibration distance for HMGTs is usually less than 3 m in practice, and the size is smaller than 50° horizontally and 30° vertically and it will not be convenient for the user to fixate on targets that have larger viewing angles. The other thing that affects the size is the hardware components that clutter user’s view (e.g. eye camera and goggles’ frame). With these considerations, it is very unlikely that a calibration pattern covers the entire scene image, thus S^cal^_int_ is usually less than 40% when using a lens with a field of view larger than 70°×50° on the scene camera. Whereas, the calibration grid usually covers more than 80% of the computer display in a remote eye tracking setup.

The number of calibration points is another important factor to consider. Manually selecting the calibration targets in the image slows down the calibration procedure and it could also affect the calibration result due to the possible head (and therefore camera) movements during the calibration. Therefore, to minimize the calibration time and accuracy, HMGTs with manual calibration often use no more than 9 calibration points. However, detecting the targets automatically allows for collecting more points in an equivalent amount of time when the user looks at a set of target points in the calibration plane or by following a moving target. Thus the practical number of points for calibration really depends on the calibration method. It might for example be worth to collect 12 or 16 points instead of 9 points if this improves the accuracy significantly.

## Evaluation of different polynomial functions

The performance of the polynomial functions derived earlier are compared to an extension of the second order polynomial model suggested by Mitsugami, Ukita, & Kidode [ 14 ] and with two models suggested by Blignaut [ 3 ] and Blignaut [ 4 ]. These models are summarized in Table 4.

**Table 4:** Summary of models tested in the simulation. Functions are shown with only their terms without coeffcients.

No.	reference	S_x_	S_y_
1	Blignaut, 2014	1, x, y, xy, x², y², x²y²	1, x, y, xy, x², y², x²y²
2	Blignaut, 2013	1, x, y, xy, x², x²y², x³, x³y	1, x, y, xy, x², y², x²y
3	Blignaut, 2014	1, x, y, xy, x², y², x²y, x³, y³, x³y	1, x, y, xy, x², x²y
4	Derived above	1, x, y, xy, x², y², x²y, xy², x²y², x³, x³y, x³y²	1, x, y, xy, y², xy²
5	Derived above	1, x, y, xy, x², y², x²y, xy², x²y², x³, x³y, x³y²	1, x, y, xy, x², y², x²y, xy², x²y², x³, x³y, x³y²

Model 5 is similar to model 4 except that it uses Eq. 3 for both S_x_ and S_y_. The scene camera was configured with the properties from Table 2. The 4×4 calibration grid was positioned 1 m from the eye and 16×16 points uniformly distributed on the scene image were used for testing.

We tested the five polynomial models using 2 interpolation ratios (20% and 50%). Besides the 4×4 calibration grid, we used a 3×3 calibration grid for polynomial model 1.

**Figure 7.**
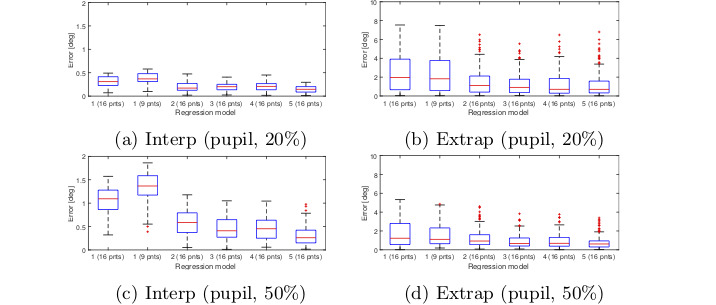
Gaze estimation error obtained from different regression models for interpolation and extrapolation regions of the scene image. Gaze estimation was based on the Pupil center and no measurement noise was applied to the eye image. Errors are measured in degrees.

The gaze estimation result for these configurations are shown in Figure 7 for the interpolation and extrapolation regions. Each boxplot show s the gaze error in a particular region measured in degrees. These figures are only meant to give an idea of how different gaze estimation functions per- form. The result shows that there is no significant difference between models 3 and 4 in the interpolation area. Increasing the calibration ratio increases the error in the interpolation region but overall gives a better accuracy for the whole image. For this test, no significant difference was observed between the models 3, 4 and 5.

**Figure 8.**
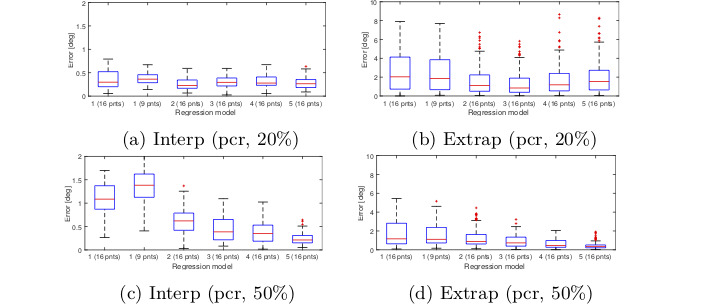
Gaze estimation error obtained from different regression models for interpolation and extrapolation regions of the scene image. Gaze estimation was based on the PCR feature and no measurement noise was applied to the eye image. Errors are measured in degrees.

Similar test was performed with pupil-cornealreflection (PCR) instead of pupil. The result for PCR condition is shown in Figure 8. The result shows that model 5 with PCR over performs other models when calibration ratio is greater than 20% even though the model was derived based on pupil position only.

To have a more realistic comparison between different models, in Section Combined Error we look at the effect of noise in the gaze estimation result by applying a measurement error on the eye image.

## Parallax Error

Assuming that the mapping function returns a precise gaze point all over the scene image, the estimated gaze point will still not correspond to the actual gaze point when it is not on the calibration plane. We refer to this error as parallax error which is due to the misalignment between the eye and the scene camera.

**Figure 9.**
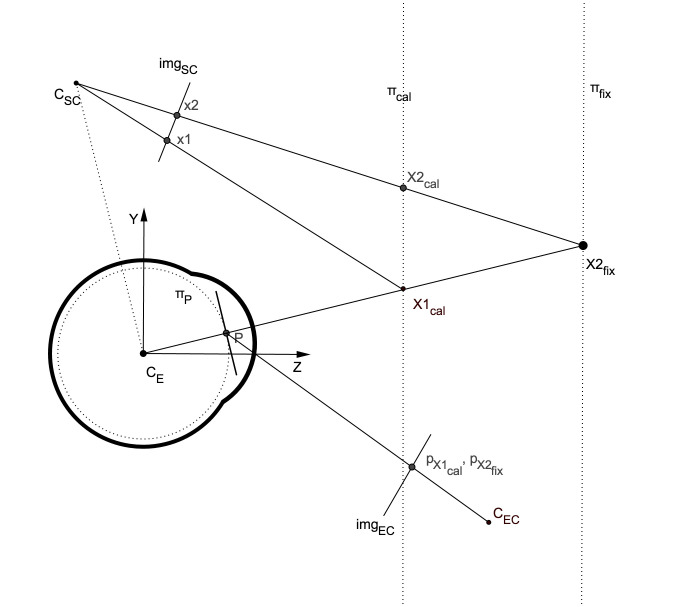
Sagittal view of a HMGT illustrating the epipolar geometry of the eye and the scene camera.

Figure 9, illustrates a head-mounted gaze tracking setup in 2D (sagittal view). It shows the offset between the actual gaze point in the image x2 and the estimated gaze point x1 when the gaze tracker is calibrated for plane π_cal_ and eye is fixating on the point X2_cal_. The figure is not to scale and for the sake of clarity the calibration and fixation planes (respectively π_cal_ and π_fix_) are placed very close to the eye. Here, the eye and scene cameras can both be considered as pinhole cameras forming a stereo-vision setup.

We define the parallax error as the vector between the actual gaze point and the estimated gaze point in the scene image (e_par_(x2) = x2x1) when the mapping function works precisely.

When the eye fixates at points along the same gaze direction, there will be no change in the eye image and consequently the estimated gaze point in the scene image remains the same. As a result, when the point of gaze (X2_fix_) moves along the same gaze direction the origin of the error vector e_par_ moves in the image, while the endpoint of the vector remains fixed.

The parallax error e_par_ for any point x in the scene image can be geometrically derived by first back-projecting the desired point onto the fixation plane (point X_fix_):

**(6):**
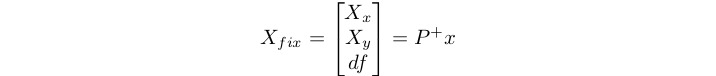


Where P^+^ is the pseudo-inverse of the projection matrix P of the scene camera. And then, intersecting the gaze vector for X_fix_ with π_cal_:

**(7):**
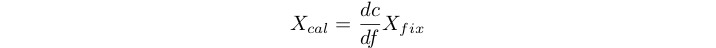


Where d_c_ is the distance from the center of the eyeball to the calibration plane and d_f_ is the distance to the fixation plane along the Z axis. Finally, projecting the point X_cal_ onto the scene camera gives us the end-point of the vector e_par_ while the initial point x in the image is actually the start-point of the vector.

By ignoring the visual axis deviation and taking the optical axis of the eye as the gaze direction, the epipole e in the scene image can be defined by projecting the center of eyeball C_E_ onto the scene image. According to epipolar geometry this can be described as:

**(8):**
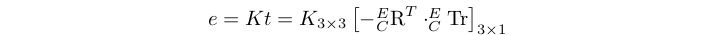


Where K is the eye camera matrix and ^E^_C_R^T^ and ^E^_C_Tr respectively rotation and translation of the scene camera related to center of the eyeball. Mardanbegi and Hansen [ 12 ] have shown that taking the visual axis deviation into account does not make a significant difference in the location of epipole in the scene image.

**Figure 10.**
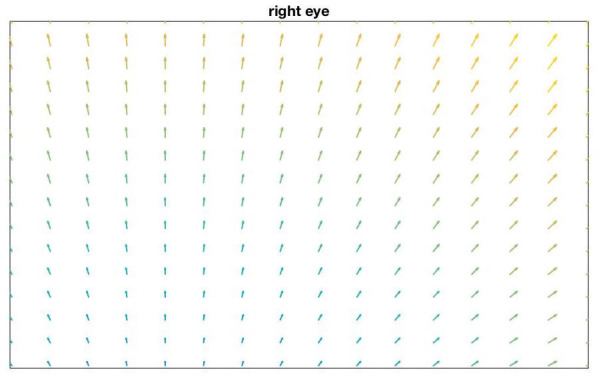
parallax error in the scene image for fixation distance at 3 m when d_cal_ = 1m on the setup described in Table2. This figure assumes an ideal mapping function with zero interpolation and extrapolation error in the entire image for the calibration distance d_cal_.

Figure 10 shows an example distribution of the parallax error in the scene image for d_cal_ = 1 m and d_fix_ = 3 m on the setup described in Table 2 when having an ideal mapping function with zero error for the calibration distance in the entire image.

## Effect of radial lens distortion

In this section we show how radial distortion in the scene image, that is more noticeable when using wideangle lenses, affects the gaze estimation accuracy in HMGT.

**Figure 11.**
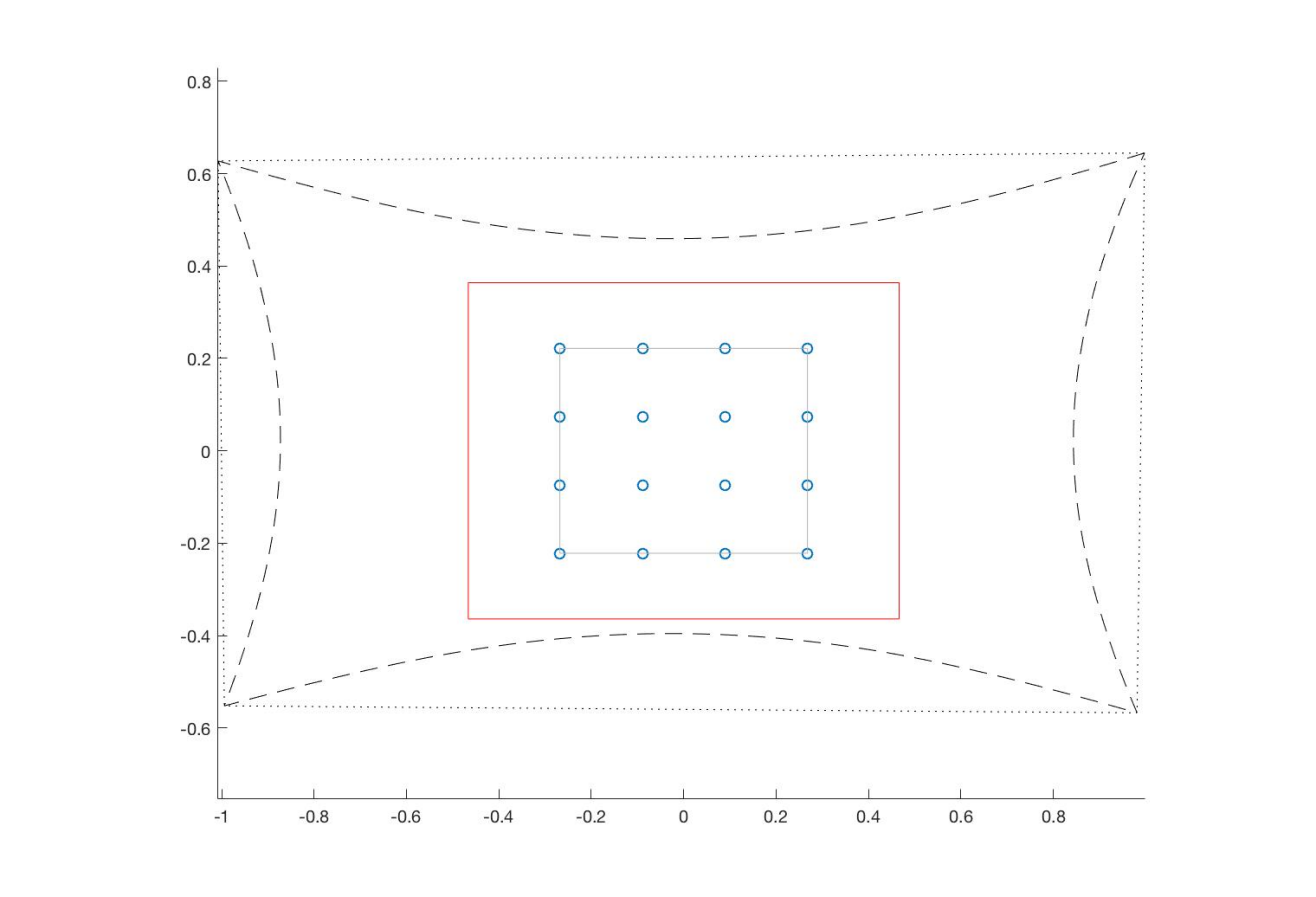
Calibration grid (small circles) and working area (red rectangle) marked in the calibration plane and borders of the scene image when it is back-projected onto the calibration plane with (dashed line) and without (dotted line) lens distortion. This figure was drawn according to the settings described in Table 5.

Figure 2 shows the location of pupil centers in the eye image when the eye fixates at points that are uniformly distributed in the scene image. These pupil-centers are obtained by back projecting the corresponding target point in the scene image onto the calibration plane, and rotating the eye optical axis towards that fixation point in the scene. When the scene image has no radial distortion, the back-projection of the scene image onto the calibration plane is shaped as a quadrilateral (dotted line in Figure 11).

**Figure 13.**
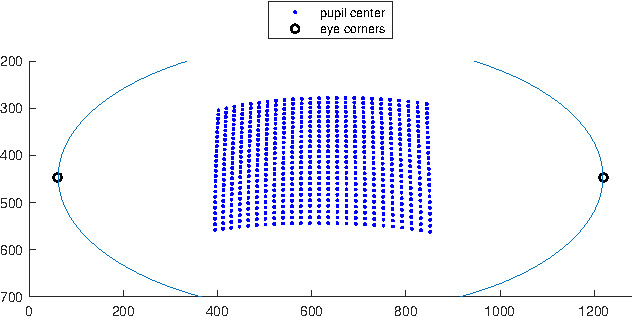
A sample eye image with pupil centers corresponding to 625 target points in the scene image when having a lens distortion.

However, when the scene image is strongly affected by radial distortion, the back-projection of the scene image onto the calibration plane is shaped as a quadrilateral with a pincushion distortion effect (dashed line in Figure 11). Figure 13 shows the corresponding pupil positions for these fixation points. By comparing Figure 13 with Figure 2, we can see that the positive radial distortion in the pattern of fixation targets caused by lens distortion, to some extent will compensate for the nonlinearity of the pupil positions and adds a positive radial distortion to the normal eye samples.

To see whether this could potentially improve the result of the regression we compared 2 different conditions one with and the other without lens distortion. We want to compare different conditions independently of the camera FoV and focal length. Since adding lens distortion to the projection algorithm of the simulation may change the FoV of the cam- era we define a “working area” which corresponds to the region where we want to have gaze estimated on. Also, a fixed calibration grid in the center of the working area is used for all conditions. Two different polynomial functions are used for gaze mapping in both conditions using the pupil center: Model 1 with a calibration grid of 3 × 3 points, and model 5 with 4 × 4 calibration points. The test is done with the parameters described in Table 5. Also, lens distortion in the simulation is modeled with a 6th order polynomial [ 19 ]:

**(9):**
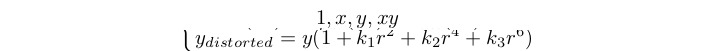


**Table 5.** Parameters used in the simulation for testing the effect of lens distortion

wide-angle lens	FoV = H : 90° × V : 60°, R = (pan, tilt, yaw) = (0, 0, 0), Tr = (10mm, 30mm, 35mm), focal length=965 pixels, distortion coeffcients= [-0.42, 0.17, -0.00124, 0.0015, -0.034], res=(1280 × 960)
calibration	FoV = H : 30° × V : 25°, calibration distance=1m
working area	FoV = H : 50° × V : 30°

**Figure 12.**
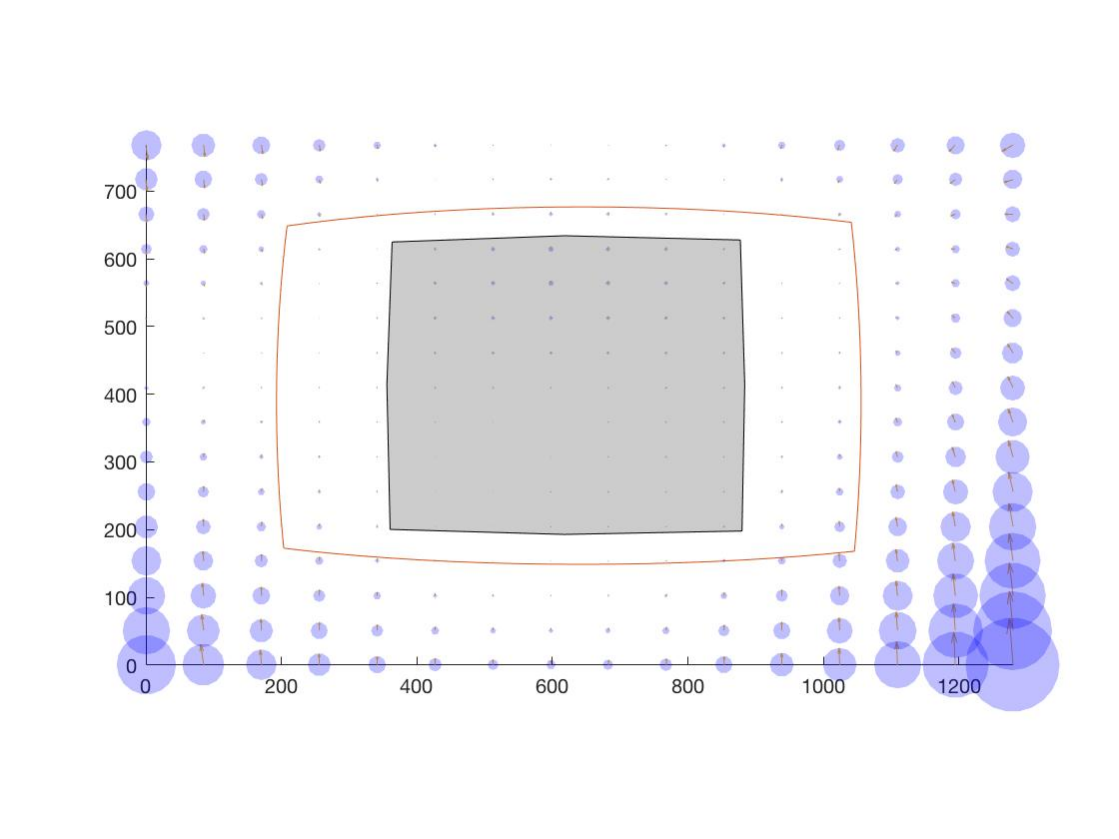
A sample image with radial distortion showing the calibration region (gray) and the working area (red curve).

Figure 12 shows a sample scene image showing the calibration and the working areas conveying the amount of distortion in the image that we get from the lens defined in Table 5.

**Figure 14.**
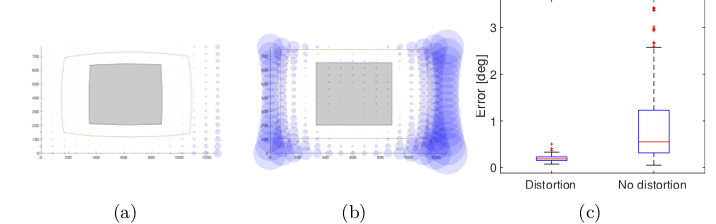
Gaze estimation error in the scene image showing the effect of radial distortion on polynomial function 1 (3 × 3 calibration points) (a) with and (b) without lens distortion. The error in the working area for both conditions is shown in (c).

**Figure 15.**
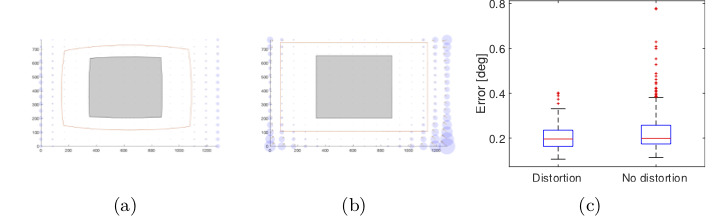
Gaze estimation error in the scene image showing the effect of radial distortion on polynomial function 5 (4 × 4 calibration points) (a) with and (b) without lens distortion. The error in the working area for both conditions is shown in (c).

Figure 14 shows a significant improvement in accuracy when having lens distortion with a second order polynomial. However, lens distortion does not have a huge impact on the performance of the model 5 (Figure 15) because this 3rd order polynomial has already compensated for the non- linearity of the pupil movements.

Besides affecting gaze mapping result, lens distortion also distorts the pattern of error vectors in the image. For example, in a condition where we have parallax error, and no error from the polynomial function, the assumption of having one epipole in the image at which all epipolar lines intersect does not hold when we have lens distortion.

## Combined Errors

In the previous sections we discussed different factors that contribute to the final vector field of gaze estimation error in the scene image. These four factors do not affect the gaze estimation independently and we cannot combine their errors by simply adding the resultant vector field of errors obtained from each. For instance, when we have e_par_ and e_int_ vector fields, the final error at point x2 in the scene image is not the sum of two e_par_(x2) and e_int_(x2) vectors. According to Figure 9, the estimated gaze point is actually Map(p_x1cal_) = x1 + e_int_(x1) which is the mapping result of pupil center p_x1cal_ that corresponds to the point x1_cal_ on π_cal_. Thus, the final error at point x2 will be:

**(10):**
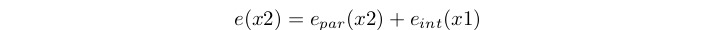


**Figure 16.**
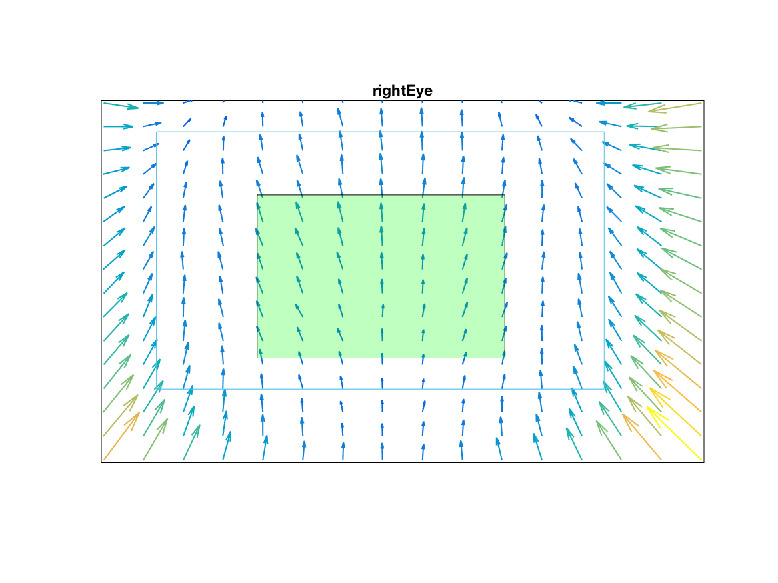
An example of error pattern in the image when having mapping error and parallax error combined.

An example error pattern in Figure 16 illustrates how much the parallax error could be deformed when it is combined with interpolation and extrapolation errors.

The impact of lens distortion factor is even more complicated as it both affects the calibration and causes a non- linear distortion in the error field. Although mathematically expressing the error vector field might be a complex task, we could still use the simulation software to generate the error vector field. This could in practice be useful if direction of the vectors in the vector field is fully defined by the geometry of the setup in HMGT. This could help manufacturers to know about the error distribution for a specific configuration which could later be used in the analysis software by weighting different areas of the image in terms of gaze estimation validity. Therefore, it will be valuable to investigate whether the error vector field is consistent and could be defined only by knowing the geometry of the HMGT.

The four main factors described in the paper are those that resulting from the geometry of different components of a HMGT system. There are other sources of error that we have not discussed such as: image resolution of both cam- eras, having noise (measurement error) in pupil tracking, pupil detection method itself, and the position of the light source when using pupil and corneal reflection. We have observed that noise and inaccuracy in detecting eye features in the eye image has the most impact in the accuracy of gaze estimation. Applying noise in the eye tracking algorithm in the simulation allows us to have a more realistic comparison between different gaze estimation functions and also shows us how much the error vectors in the scene image are affected by inaccuracy in the measurement both in terms of magnitude and direction. We did the same comparison between different models that was done in the evaluation section, but this time with two levels of noise with a Gaussian distribution (mean=0, standard deviation=0.5 and 1.0 pixel).

**Figure 18.**
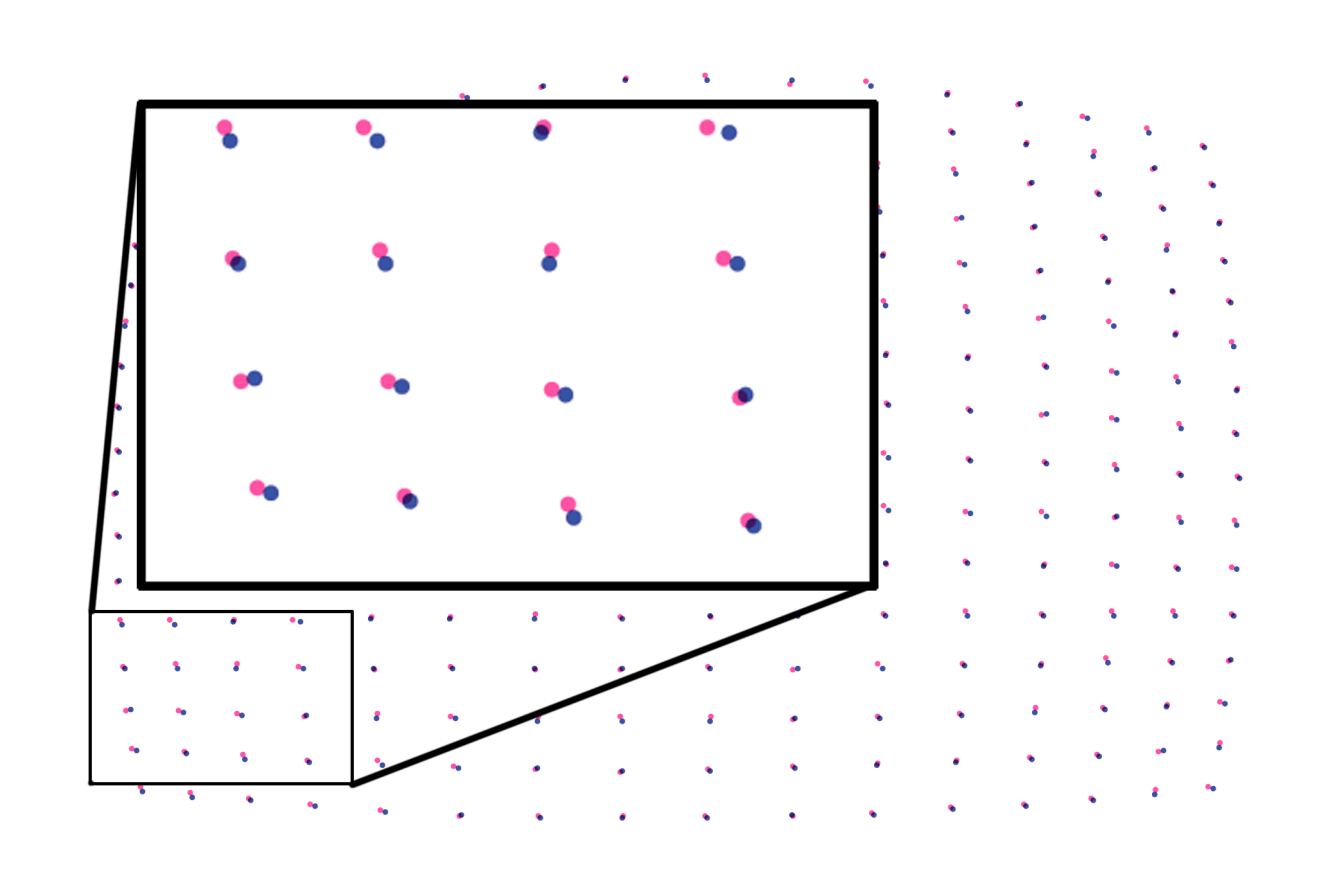
Pupil centers in the eye image corresponding to a grid of 16 × 16 fixation points on the calibration plane, are shown by red for noisy condition, and blue for without noise. Image resolution is 1280 × 960.

Figure 18 shows how much the pupil detection in the image (1280 × 960) gets affected by noise level 0.5 in the measurement. Pupil centers in the eye image corresponding to a grid of 16 × 16 fixation points on the calibration plane, are shown in red for the condition with noise, and blue for the condition without noise.

**Figure 17.**
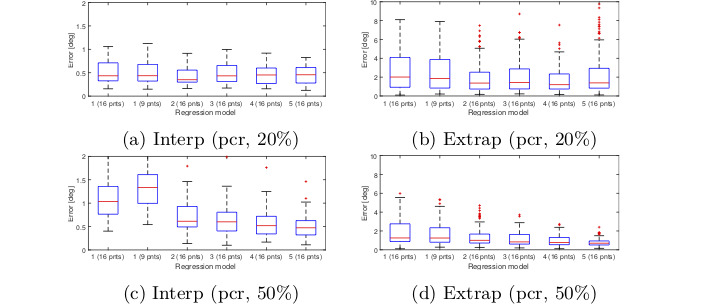
Gaze estimation error obtained from different polynomial models. Gaze estimation was based on the PCR feature. Resolution of the eye image is set to 1280 × 960 and a noise level of 0.5 is applied. Errors are measured in degrees of visual angle.

Figure 17 shows the gaze estimation result for noise level 0.5 with PCR method. No radial distortion was included and the noise was added both during and after the calibration. The result shows how the overall error gets lower when increasing the calibration ratio from 20% to 50%.

**Figure 19.**
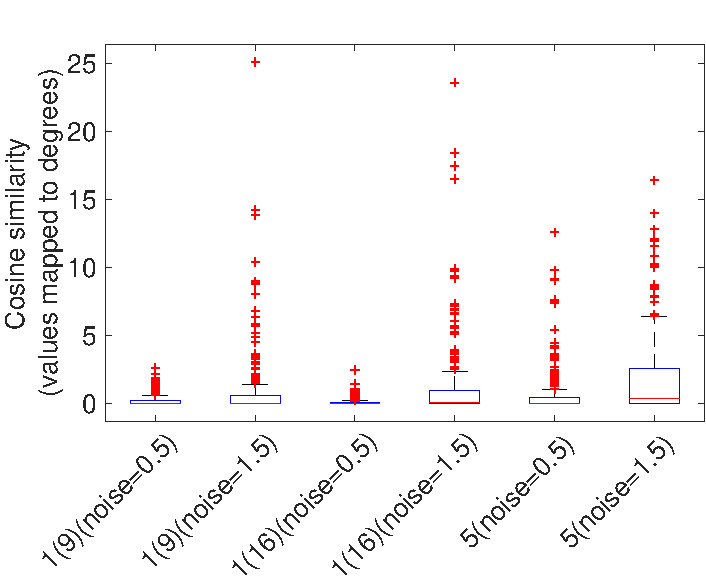
This figure shows how much different levels of measurement noise (in model 1 and 5) affects the direction of error vectors when having parallax error. The vertical axis represents the angular deviation.

To see the impact of noise on the direction of vectors in the image, a cosine similarity measure is used for comparing the two vector fields (each containing 16 × 16 vectors). We compare the vector fields obtained from 2 different noise levels (0.5 and 1.5) with the vector field obtained from the condition with no noise. For this test, the calibration and the fixation distances are respectively set to 0.7 m and 3 m. Adding parallax error makes the vector field more meaningful in the no-noise condition. In this comparison we ignore the differences in magnitude of the error and only compare the direction of vectors in the image. Figure 19 shows how much, direction of vectors deviates when having measurement noise in practice with model 1 and 5.

Based on the results shown in Figure 17 we can conclude that we get almost the same gaze estimation error in the interpolation region for all the polynomial functions. Having too much noise, has a great impact on the magnitude of the error vectors in the extrapolation region and the effect is even greater in the 3rd order polynomial models. Figure 19 indicates that despite the changes in the magnitude of the vectors, when having noise, direction of the vectors does not change significantly. This means that the vector field obtained based on the geometry could be used as a reference for predicting at which parts of the scene image the error is larger (relative to the other parts) and how the overall pattern of error would be. However, this needs to be validated empirically on real HMGT.

**Figure 20.**
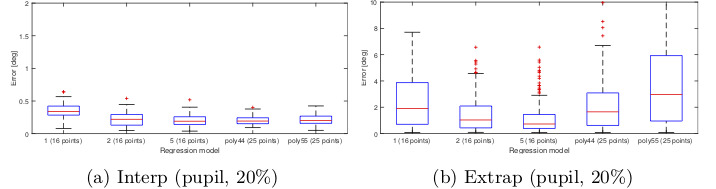
Comparing the performance of higher order polynomials (4th and 5th order) with 25 calibration points with 3rd order polynomial models using 16 points for calibration

Another test was conducted to check the performance of higher order polynomial models. The test was done with calibration ratio of 20% and noise level 0.5 using a pupil-only method. A 4 × 4 calibration grid was used for models 1, 2 and 5 and a 5 × 5 grid for 4th and 5th order standard polynomial models. The gaze estimation result of this comparison is shown in Figure 20 confirming that performance does not improve with higher order polynomial models even with more calibration points.

## Conclusion

In this paper we have investigated the error distribution of polynomial functions used for gaze estimation in head-mounted gaze trackers (HMGT). To describe the performance of the functions we have characterized four different sources of error. The interpolation error is measured within the bounding box defined by the calibration points as seen by the scene camera. The extrapolation error is measured in the remaining area of the scene camera outside the calibration bounding box. The other two types of error are due to the parallax between the scene camera and the eye, and the radial distortion of the lens used in the scene camera. Our results from simulations show that third order polynomials provide better overall performance than second order and even higher order polynomial models.

We didn’t find any significant improvement of model 5 over model 4, especially when the noise is present in the input (comparing figures 17 and 8). This means that it’s not necessary to use higher order polynomials for S_y_.

Furthermore, we have shown that using wide angle lens scene cameras actually reduces the error caused by non- linearity of the eye features used for gaze estimation in HMGT. This could improve the results of the second order polynomial models significantly as these models suffer more from the non-linearity of the input. Although the 3rd order polynomials provide more robust results with and without lens distortion, the 2nd order models have the advantage of requiring fewer calibration points. We replicated the same analysis we did for deriving model 4 but with the effect of radial distortion in the scene image. We found linear relationships between S_x_ and P_x_ and also between S_y_ and P_y_. The relationship between S and the coefficients were also linear suggesting the following model for both S_x_ and S_y_:

**(11):**
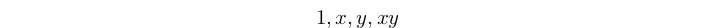


As a future work we would like compare the performance of the models discussed in this paper on a real head-mounted eye tracking setup and see if the results obtained from the simulation could be verified. It would also be interesting to compare the performance of a model based on Eq.11 on a wide angle lens with model 4 on a non-distorted image. The simulation shows that the gaze estimation accuracy obtained from a model based on Eq.11 with 4 calibration points on a distorted image is as good as the accuracy obtained from model 4 with 16 points on a non-distorted image. This, however, needs to be verified on a real eye tracker.

Though an analytical model describing the behavior of the errors might be feasible, the simulation software developed for this investigation might help other researchers and manufacturers to have a better understanding of how the accuracy and precision of the gaze estimates vary over the scene image for different configuration scenarios and help them to define configurations (e.g. different cameras, lenses, mapping functions, etc) that will be more suitable for their purposes.
